# Exogenous Substrates Prevent the Decline in the Cellular ATP Content of Primary Rat Astrocytes During Glucose Deprivation

**DOI:** 10.1007/s11064-024-04104-0

**Published:** 2024-02-11

**Authors:** Antonia Regina Harders, Paul Spellerberg, Ralf Dringen

**Affiliations:** 1 Centre for Biomolecular Interactions Bremen, Faculty 2 (Biology/Chemistry), University of Bremen, P.O. Box 330440, 28334 Bremen, Germany; 2 Centre for Environmental Research and Sustainable Technologies, University of Bremen, Bremen, Germany

**Keywords:** Amino acids, ATP, Fatty acids, Metabolism, Mitochondria, Nucleosides, Proline

## Abstract

Brain astrocytes are well known for their broad metabolic potential. After glucose deprivation, cultured primary astrocytes maintain a high cellular ATP content for many hours by mobilizing endogenous substrates, but within 24 h the specific cellular ATP content was lowered to around 30% of the initial ATP content. This experimental setting was used to test for the potential of various exogenous substrates to prevent a loss in cellular ATP in glucose deprived astrocytes. The presence of various extracellular monocarboxylates, purine nucleosides or fatty acids prevented the loss of ATP from glucose-deprived astrocytes. Of the 20 proteinogenic amino acids, only alanine, aspartate, glutamate, glutamine, lysine or proline maintained high ATP levels in starved astrocytes. Among these amino acids, proline was found to be the most potent one to prevent the ATP loss. The astrocytic consumption of proline as well as the ability of proline to maintain a high cellular ATP content was prevented in a concentration-dependent manner by the proline dehydrogenase inhibitor tetrahydro-2-furoic acid. Analysis of the concentration-dependencies obtained by considering the different carbon content of the applied substrates revealed that fatty acids and proline are more potent than glucose and monocarboxylates as exogenous substrates to prevent ATP depletion in glucose-deprived astrocytes. These data demonstrate that cultured astrocytes can utilise a wide range of extracellular substrates as fuels to support mitochondrial ATP regeneration and identify proline as potent exogenous substrate for the energy metabolism of starved astrocytes.

## Introduction

Brain astrocytes have a very broad metabolic potential and are known to use endogenous and exogenous sources as substrates for their energy metabolism [1, 2]. Extensive knowledge has been accumulated during the last decades on the astrocytic metabolism of carbohydrates [2–4], the astrocytic mitochondrial metabolism [5], the metabolism of amino acids such as glutamate and glutamine [6], the fatty acid metabolism [7, 8] as well as their nucleotide metabolism [9, 10]. Although brain astrocytes are considered to be rather glycolytic cells [5, 11, 12], astrocytes at least in culture regenerate most of their ATP by oxidative phosphorylation [13]. During glucose-deprivation, cultured astrocytes maintain a high cellular ATP concentration of around 7 mM for at least 8 h by using endogenous energy stores such as fatty acids as substrates to fuel ATP regeneration [13]. However, also several external substrates have been reported to be metabolized in glucose-deprived cultured astrocytes to prevent ATP depletion including monocarboxylates [13], hexoses such as mannose and fructose [13] and purine nucleosides [14].

Additional extracellular substrates that have to be considered as extracellular energy substrates for mitochondrial ATP regeneration in astrocytes are fatty acids and amino acids. The potential of astrocytes to metabolise exogenous fatty acids by mitochondrial β-oxidation and subsequent oxidation to CO_2_ or ketogenesis has been reported for cultured astrocytes [15–19] and for astrocytes in brain slices [20, 21]. In addition, astrocytes have been shown to contribute to the removal of neuron-derived toxic fatty acids [22, 23].

Among the proteinogenic amino acids that may be efficiently metabolised by astrocytes to provide energy by mitochondrial oxidation of their carbon skeleton, especially alanine, glutamate and aspartate have to be considered as their transamination leads directly to the α-ketoacids pyruvate, α-ketoglutarate and oxaloacetate, respectively. The latter two are intermediates of the citric acid cycle, while pyruvate is substrate of both pyruvate dehydrogenase and pyruvate carboxylate which provide acetyl-CoA and oxaloacetate as substrates of the citrate synthase. Indeed, rapid uptake and metabolism by cultured astrocytes has been reported and discussed for alanine [24, 25], glutamate [6, 26] and aspartate [27, 28].

During glucose-deprivation of cultured astrocytes for 24 h, the viability of the cells was not compromised although the specific cellular ATP content had been lowered by around 70% [13]. This experimental paradigm of a 24 h glucose deprivation was applied in the current study to investigate the potential of cultured astrocytes to use a wide range of exogenous substrates as potential fuels to maintain a high cellular ATP content during glucose starvation. The data obtained reveal that glucose-deprived cultured astrocytes possess the capacity to take up and efficiently metabolize in concentration-dependent manners various exogenous substrates for mitochondrial ATP regeneration to maintain a high cellular ATP content, including monocarboxylates, selected amino acids, fatty acids as well as purine nucleosides. Correlation of the concentration-dependencies of the ATP maintaining effects of the investigated exogenous substrates to their content of oxidisable carbon atoms revealed that fatty acids and proline are more potent than exogenous glucose or monocarboxylates to maintain a high cellular ATP content in glucose-deprived astrocytes.

## Materials and Methods

### Materials and Chemicals

Sterile cell culture materials, unsterile 96-well plates and black microtiter plates were obtained from Sarstedt (Nümbrecht, Germany). Dulbecco’s modified Eagles medium powder (DMEM with 25 mM glucose) and penicillin G/streptomycin sulfate solution were from Thermo Fisher Scientific (Schwerte, Germany; RRID:SCR_008452). Fetal calf serum (FCS), the three fatty acids applied, alanine, glutamine, glutamate, isoleucine, lysine, serine, threonine, tryptophan, glucose, lactate, adenosine, inosine, guanosine, and dipyridamole were purchased from Sigma-Aldrich (Steinheim, Germany; RRID:SCR_008988). Bovine serum albumin (BSA), dimethyl sulfoxide (DMSO), perchloric acid, NAD^+^, NADH, arginine, histidine, leucine, methionine, tyrosine and valine were from AppliChem (Darmstadt, Germany; RRID:SCR_005814). Adenosine triphosphate (ATP) was purchased from Roche Diagnostics (Mannheim, Germany; RRID:SCR_001326) and adenosine diphosphate (ADP) from Acros Organics (New Jersey, USA). Asparagine, aspartate, cysteine, phenylalanine, proline, glacial acetic acid and adenosine monophosphate (AMP) were obtained from Fluka (Buchs, Switzerland). Fatty acid-free BSA was from Capricorn Scientific (Ebsdorfergrund, Germany), etomoxir (HY-50202) and ninhydrin were from Merck (Darmstadt, Germany; RRID:SCR_001287). 4-(2-Hydroxyethyl) piperazine-1-ethanesulfonic acid (HEPES) was purchased from Carl Roth (Karlsruhe, Germany). The Cell Titer Glo® 2.0 ATP Assay Kit (G9241) was from Promega (Walldorf, Germany; RRID:SCR_006724). Tetrahydro-2-furoic acid (THFA) (SC-253674) was obtained from Santa Cruz Biotechnology (Heidelberg, Germany).

### Astrocyte Cultures

Primary astrocyte-rich cultures were prepared from the brains of newborn Wistar rats as previously described [29]. The rats had been purchased from Charles River Laboratories (Sulzfeld, Germany; RRID:SCR_003792) and were treated in accordance to the German and European animal welfare acts. The cells harvested from the rat brains were suspended in culture medium (90% DMEM containing 25 mM glucose, 44.6 mM sodium bicarbonate, 1 mM pyruvate, 20 U/mL penicillin G, 20 µg/mL streptomycin sulfate, supplemented with 10% FCS) to a cell density of 300,000 viable cells/mL and 1 mL of this cell suspension was seeded into the wells of 24-well dishes. The cultures were incubated in a humidified atmosphere containing 10% CO_2_ in a Sanyo CO_2_ incubator (Osaka, Japan). The culture medium was renewed every seventh day and one day prior to experiments. The data presented here were obtained on cultures of an age between 14 and 28 days. In this range, the specific ATP content was not affected by the culture age [13, 30]. Astrocyte-rich primary cultures contain mainly glial fibrillary acidic protein-positive astrocytes and only low numbers of contaminating other types of glial cells [29, 31, 32]

### Experimental Incubation of the Cells

The cultures were washed twice with 1 mL pre-warmed (37 °C) glucose- and amino acid-free incubation buffer (IB; 145 mM NaCl, 20 mM HEPES, 5.4 mM KCl, 1.8 mM CaCl_2_, 1 mM MgCl_2_, 0.8 mM Na_2_HPO_4_, pH adjusted with NaOH to 7.4 at 37 °C) and incubated for 24 h in 250 µL glucose-free IB that had been supplemented with the given exogenous substrates and/or inhibitors of transporters or enzymes. After 24 h of incubation at 37 °C in the humidified atmosphere of a CO_2_-free incubator the incubation media were harvested for determination of extracellular lactate dehydrogenase (LDH) activity, while the cells were washed twice with 1 mL ice-cold (4 °C) phosphate-buffered saline (PBS; 10 mM potassium phosphate buffer pH 7.4 containing 150 mM NaCl) and lysed for ATP quantification.

For experiments regarding the potential of fatty acids as extracellular energy substrates, the fatty acids were applied in protein-bound form [33] to improve accessibility of these lipophilic compounds to the cells. Briefly, fatty acids (pre-dissolved in ethanol as 100-fold concentrated stock solutions) were mixed with IB containing fatty acid-free BSA in a final concentration of 13.2 mg/mL and incubated at 50 rpm for 24 h at room temperature on a shaker (DUOMAX 1030, Heidolph Schwabach, Germany). Subsequently, the preincubated incubation media containing the protein-bound fatty acids were applied to the cells. Etomoxir and dipyridamole were dissolved as 1000-fold concentrated stock solutions in DMSO. Control incubations with solvents revealed that the presence of DMSO in the final concentrations applied (up to 1%), does not affect cell viability nor the cellular ATP content of treated cells under the conditions used [13].

### Quantification of Cellular ATP Content

The cellular ATP content of cultured astrocytes before and after a given treatment was determined in perchlorate lysates by a luciferin-luciferase-based luminometric assay with the Cell Titer Glo® 2.0 ATP Assay Kit in microtiter plate format as recently described in detail [13, 25] using ATP standards in a concentration range of up to 1000 nM.

### Determination of Cell Viability and Protein Content

The toxic potential of a given treatment was determined by measuring the extracellular activity of the cytosolic enzyme LDH that is released from damaged cells as described previously in detail [29]. The LDH activity determined for 10 µL samples of incubation media was compared with the initial cellular LDH activity to determine the percental toxicity. The initial cellular protein content of the cultures was determined by the Lowry method [34] using BSA as standard protein.

### Determination of Extracellular Proline Concentration

The extracellular proline concentrations after a 24 h incubation of cultured astrocytes with proline was determined by a modification of a colorimetric assay that uses the formation of a specific proline-ninhydrin condensation product under acidic conditions at high temperature [35]. Briefly, samples of IB that had been collected after the incubation of cells with proline were diluted with IB. 100 µL of the dilution (or of proline standards in IB in concentrations between 0 and 200 µM) were mixed with 200 µL of glacial acetic acid containing 1.25% (w/v) ninhydrin and subsequently incubated at 100 °C for 30 min in a thermoblock (MBT 250, Kleinfeld Labortechnik, Gehrden, Germany). After cooling to room temperature, 200 µL of the reaction mixtures were transferred to wells of a microtiter plate and the absorbance of the generated proline-ninhydrin condensation product was determined at 514 nm in a microtiter plate photometer (Multiscan sky, Thermo Fisher Scientific, Schwerte, Germany). Proline concentrations in samples were calculated by making use of the linear calibration curve generated from the absorbances obtained for the proline standards.

### Data Presentation and Statistical Analysis

The data shown in the figures and tables represent means ± standard deviations (SD) of values that had been obtained in three or more (n) experiments that were each performed in duplicates on independently prepared astrocyte cultures. Analysis for statistical significance between groups of data was performed by ANOVA (followed by the Bonferroni post-hoc test) and between two sets of data by a t-test. Levels of significance of data compared to the data obtained for the indicated control condition are given as *^,#^p < 0.05, **^,##^p < 0.01 and ***^,###^p < 0.001. p > 0.05 was considered as not significant.

## Results

### ATP Contents of Cultured Astrocytes After 24 h Incubation with or Without Glucose

**Fig. 1:**
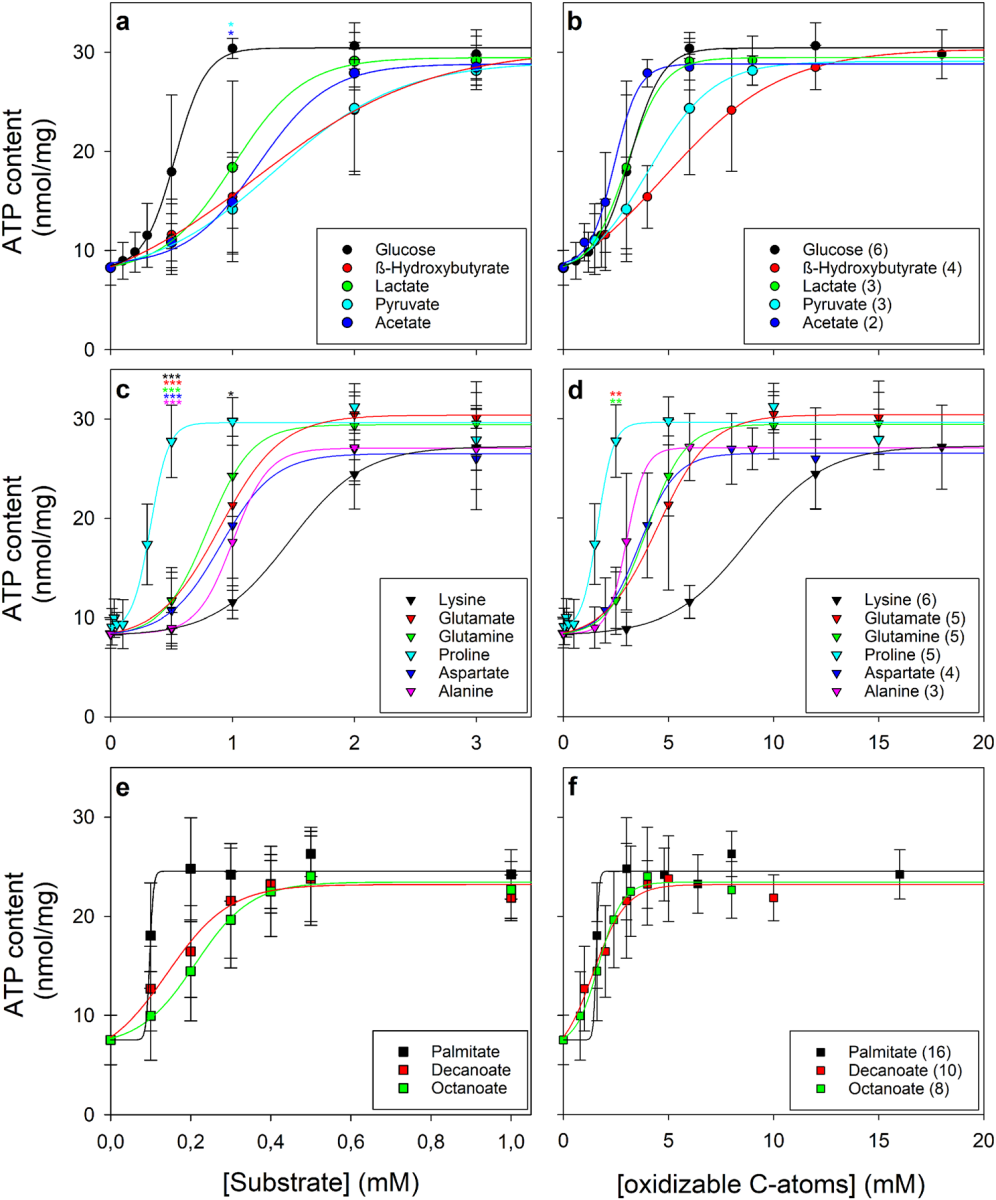
Ability of different extracellular substrates to maintain a high ATP level in glucose-deprived cultured astrocytes. The cultures were incubated for 24 h in glucose-free incubation buffer that had been supplemented by the given molar concentrations of extracellular substrates (**a**, **c**, **e**) before the specific cellular ATP content was determined. In addition, the specific cellular ATP contents were presented as function of the applied concentrations of oxidisable carbon atoms (see Table 2) of the respective substrates (**b**, **d**, **f**). The initial cellular ATP contents of the cultures used were 32.6 ± 3.5 (**a**, **b**), 35.3 ± 7.2 (**c**, **d**) and 31.9 ± 3.2 (**e**, **f**) nmol/mg and the initial protein contents were 120 ± 15 (**a**, **b**), 140 ± 22 (**c**, **d**) and 113 ± 4 (**e**, **f**) µg/well. None of the conditions used caused any significant increase in the extracellular LDH activity compared to the respective control condition (3 mM glucose; data not shown). The data represent means ± SD of values that were obtained in experiments performed on 3 independently prepared cultures. The significance of differences (ANOVA with Bonferroni post hoc test) compared with the data obtained for glucose (**a**, **b**), proline (**c**, **d**) and palmitate (**e**, **f**) is indicated by *p < 0.05, **p < 0.01 and ***p < 0.001 in the colours of the symbols representing the given substrates

The specific ATP content of cultured astrocytes has been reported to be lowered by a 24 h incubation in a glucose- and amino acid-free incubation buffer [13]. To confirm this finding and to define the basal glucose-free control conditions we analysed experimental data from a total of 39 experiments that had been performed on 30 independently prepared cultures. The initial specific ATP content of cultured astrocytes (30 ± 5 nmol/mg) was lowered during a 24 h incubation in the absence of glucose by 72% to 8 ± 2 nmol/mg, while in the presence of 3 mM glucose 87 ± 13% of the initial specific ATP contents were maintained (Table 1). None of these incubation conditions caused cell toxicity as demonstrated by the very low extracellular LDH activities determined after the 24 h incubations (Table 1). Analysis of the concentration dependent potential of glucose to maintain ATP levels during a 24 h incubation revealed half-maximal and maximal ATP maintaining effects at initial glucose concentrations of around 0.5 mM (Table 2) and 1 mM (Fig. 1a), respectively.

**Table 1:** ATP content of glucose-deprived and glucose-fed astrocytes

	Incubation time (h)	ATP content (nmol/mg)	Extracellular LDH activity (%)	n
Untreated	0	30 ± 5	0	39
No glucose	24	8 ± 2***	8 ± 8	39
3 mM glucose	24	26 ± 4*	5 ± 5	23

Cultured primary astrocytes were incubated for 24 h in glucose- and amino acid-free incubation buffer in the absence (No glucose) or the presence of 3 mM glucose. Cellular ATP content and cellular LDH activity for untreated cells (before the incubation) and the cellular ATP content and the extracellular LDH activity (in % of the initial cellular LDH activity) after the 24 h incubations were determined. The initial cellular LDH activity at the onset of the incubation was 167 ± 43 nmol/(min × well). The data represent mean values ± SD obtained in 23 and 39 individual experiments performed on 17 and 30, respectively, independently prepared cultures. The significance of differences (ANOVA with Bonferroni post hoc test) compared with the data obtained for untreated cells is indicated by *p < 0.05 and ***p < 0.001

**Table 2:** Comparison of the potential of exogenous substrates to maintain a high cellular ATP content in glucose-deprived astrocytes

Substrate	EC50	Number of oxidisable carbon atoms	EC50 x number of oxidisable carbon atoms	ATP content maintained by the highest substrate concentration applied			n
	(µM)		(mM)	(mM)	(nmol/mg)	(% of initial)	
Glucose	554 ± 103	6	3.33 ± 0.62	3	27.8 ± 3.4	83 ± 10	9
β-Hydroxybutyrate	1166 ± 117^***^	4	4.66 ± 0.47	3	28.5 ± 2.3	87 ± 7	3
Lactate	954 ± 361^**^	3	2.33 ± 0.90	3	29.3 ± 1.8	86 ± 5	6
Pyruvate	1010 ± 183^*^	3	3.03 ± 0.55	3	28.2 ± 1.5	86 ± 5	3
Acetate	1196 ± 281^***^	2	2.39 ± 0.56	3	28.5 ± 1.3	87 ± 4	3
Palmitate	103 ± 13^*^	16	1.64 ± 0.21^**^	1	24.2 ± 2.5	76 ± 8	3
Decanoate	157 ± 83	10	1.57 ± 0.83^**^	1	21.9 ± 2.3	69 ± 7	3
Octanoate	214 ± 56	8	1.71 ± 0.45^**^	1	22.7 ± 2.9	71 ± 9	3
Lysine	1603 ± 70 ^***, +++^	6	9.62 ± 0.42 ^***, +++^	3	27.1 ± 4.2	77 ± 12	3
Glutamate	909 ± 44 ^+++^	5	4.55 ± 0.22 ^+++^	3	30.1 ± 3.7	85 ± 10	3
Glutamine	778 ± 15 ^+++^	5	3.89 ± 0.08 ^+++^	3	29.5 ± 3.1	84 ± 9	3
Proline	323 ± 7	5	1.62 ± 0.04^**^	3	27.9 ± 3.1	79 ± 9	3
Aspartate	842 ± 27 ^+++^	4	3.37 ± 0.11 ^+++^	3	26.0 ± 5.1	74 ± 14	3
Alanine	1010 ± 29 ^*, +++^	3	3.03 ± 0.09 ^+++^	3	27.0 ± 2.1	76 ± 6	3

The initial concentrations of exogenous substrates that allowed half-maximal (EC50) maintenance of cellular ATP contents during a 24 h incubation was calculated for the total number of concentration-dependencies performed for the indicated substrates. In addition, the ATP contents determined for the given concentration of a substrate was included as specific content (nmol ATP/mg) and as percent of the initial ATP content at the onset of the 24 h incubation. The significance of differences (ANOVA with Bonferroni post hoc test) compared with the data obtained for glucose-treated cells is indicated by *p < 0.05, **p < 0.01 and ***p < 0.001. For amino acid-treated cells, the significance of differences compared to a proline-treatment is indicated by ^+++^p < 0.001

### ATP Maintenance by Exogenous Monocarboxylates in Glucose-Deprived Astrocytes

Lactate, pyruvate, acetate and β-hydroxybutyrate have previously been reported to prevent in a high concentration of 5 mM the ATP depletion during a 24 h incubation of glucose-deprived astrocytes [13]. The investigation of the concentration-dependency of the ATP maintaining effects revelated that the four monocarboxylates investigated had a similar potential to prevent ATP loss in glucose-deprived astrocytes (Fig. 1a) with half-maximal and maximal ATP maintaining effects found for concentrations of around 1 mM (Table 2) and 2 mM (Fig. 1a), respectively. Considering the half-maximal effect concentrations of the substrates applied, the monocarboxylates were found less potent to prevent the loss in cellular ATP content compared to glucose (Fig. 1a; Table 2). However, it should be considered that glucose and the investigated monocarboxylates contain different numbers of carbon atoms that can be oxidised to provide energy by mitochondrial metabolism. Taking the number of oxidisable carbon atoms of the applied exogenous substrates into account, the concentration-dependencies of the ATP maintaining effects were shifted (Fig. 1b) and the concentrations calculated for the monocarboxylates and for glucose to provide half-maximal ATP maintaining potential were not significantly different anymore (Table 2).

### ATP Maintenance by Exogenous Amino Acids in Glucose-Deprived Astrocytes

**Fig. 2:**
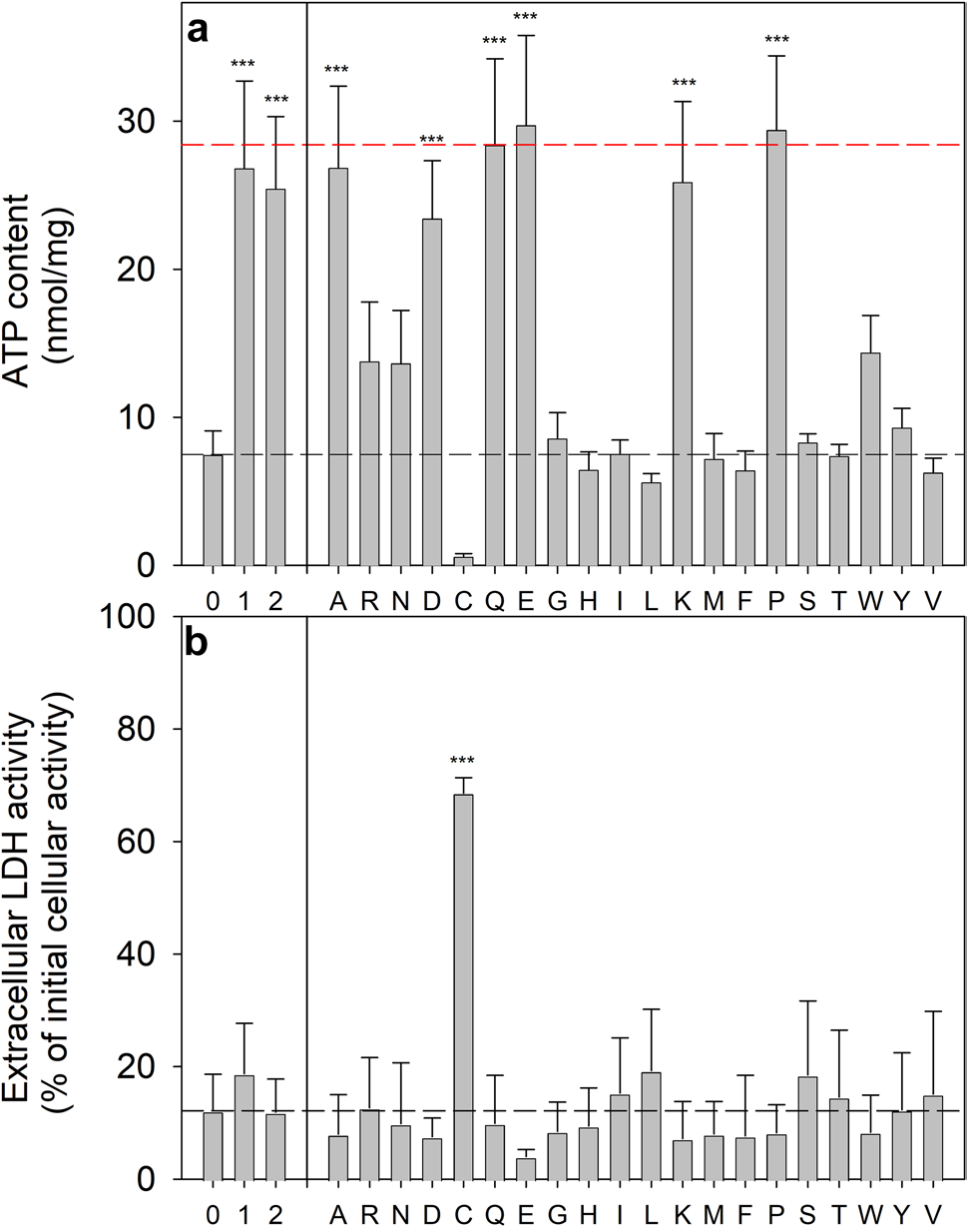
Maintenance by amino acids of a high ATP level in glucose-deprived cultured astrocytes. The cultures were incubated for 24 h in glucose-free incubation buffer (0) or in buffer that had been supplemented with 3 mM of glucose (1), lactate (2) or the indicated amino acids (in one letter code) before the specific cellular ATP content (**a**) and the extracellular LDH activity (**b**) were determined. The initial cellular ATP content at the onset of the incubation (dashed red line) was 28.4 ± 5.6 nmol/mg, the initial cellular LDH activity was 198 ± 38 nmol/(min × well) and the initial protein content was 144 ± 10 µg/well. The data represent means ± SD of values that were obtained in experiments performed on 3 independently prepared cultures. The significance of differences (ANOVA with Bonferroni post hoc test) compared with the data obtained for cells that had been incubated without glucose and amino acids (0) are indicated by ***p < 0.001

In order to test for the potential of amino acids to serve as extracellular fuels to maintain a high cellular ATP content during glucose deprivation, cultured astrocytes were incubated in glucose-free buffer in the presence of one of the 20 proteinogenic amino acids for 24 h. Of the conditions applied, only the treatment with cysteine severely compromised the cell viability as demonstrated by the absence of detectable ATP in the cells (Fig. 2a) and by the high extracellular LDH activity (Fig. 2b). For all other amino acids investigated, no obvious cell toxicity was observed (Fig. 2b). The decline in the specific ATP content during incubation in glucose-free buffer for 24 h was prevented by supplementation of the buffer with 3 mM of either alanine (A), aspartate (D), glutamate (E), glutamine (Q), lysine (K) or proline (P) (Fig. 2a), while the presence of one of the other amino acids investigated did not show any ATP maintaining potential (Fig. 2a). In contrast to proline, 4-hydroxyproline in a concentration of 3 mM was unable to prevent the loss in cellular ATP of glucose-deprived astrocytes (data not shown).

Investigation of the concentration-dependency of the ATP maintaining potential of the six amino acids that have been identified as exogenous fuels for ATP regeneration (Fig. 2a) revealed that proline was the most potent among those amino acids (Fig. 1c; Table 2). The presence of glutamate, glutamine, aspartate or alanine had similar potentials to prevent ATP loss in glucose-deprived astrocytes, while lysine had to be applied in higher concentrations than the other amino acids to serve as efficient fuel for ATP regeneration (Fig. 1c; Table 2). The high potential of proline, compared to other amino acids, to maintain a high cellular ATP content in glucose-deprived astrocytes is supported by the significantly lower half-maximal ATP-maintaining concentration of proline (Table 2). Considering the number of carbon atoms of the applied exogenous substrates, proline appears to be even better than glucose and monocarboxylates to prevent ATP loss in glucose-deprived astrocytes (Fig. 1b, d; Table 2).

**Fig. 3:**
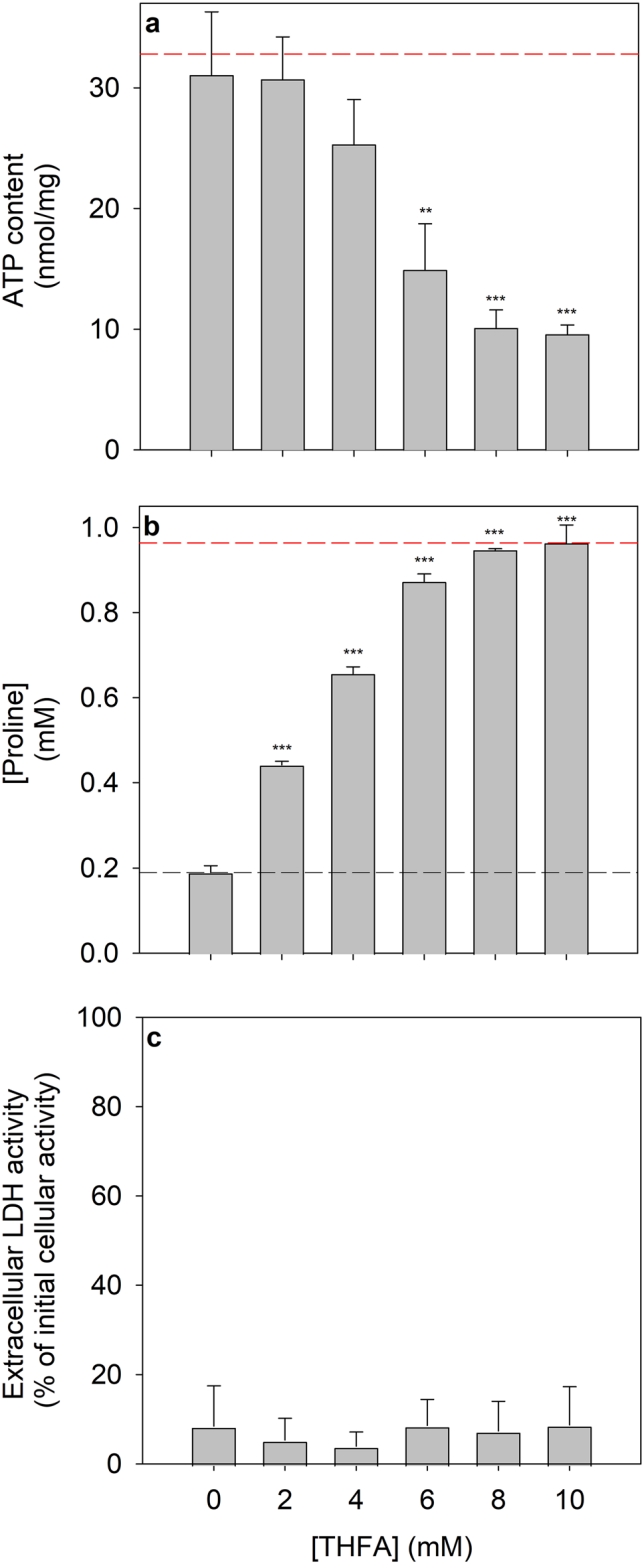
Consequences of an application of THFA on the ATP content and the extracellular proline concentration of glucose-deprived cultured astrocytes. The cultures were incubated for 24 h in glucose-free incubation buffer containing proline in an initial concentration of 1 mM in the absence or the presence of the given concentrations of the proline dehydrogenase inhibitor THFA before the specific cellular ATP content (**a**), the extracellular proline concentration (**b**) and the extracellular LDH activity (**c**) were determined. The initial cellular ATP content at the onset of the incubation was 32.8 ± 4.5 nmol/mg, the initial cellular LDH activity was 181 ± 38 nmol/(min × well) and the initial protein content was 129 ± 7 µg/well. The data shown represent means ± SD of values that were obtained in experiments performed on 3 independently prepared cultures. The significance of differences (ANOVA with Bonferroni post hoc test) compared with the data obtained for cells that had been incubated without the inhibitor (0 mM) are indicated by **p < 0.01 and ***p < 0.001

After exposure of glucose-deprived astrocytes to 1 mM proline, the ATP content of the cells was maintained high (Fig. 3a), while around 0.8 mM of the initially applied proline had been consumed by the cells (Fig. 3b). The ability of proline to maintain a high cellular ATP level (Fig. 3a) as well as the potential of the cells to consume applied proline (Fig. 3b) was impaired in a concentration-dependent manner by the presence of THFA, a competitive inhibitor of the proline dehydrogenase [36]. At a THFA concentration of 8 mM, proline consumption (Fig. 3b) as well as the utilization of proline to maintain a high cellular ATP content of starved astrocytes (Fig. 3a) were completely prevented. None of the conditions investigated caused any obvious impairment of cell viability as indicated by the absence of any increase in extracellular LDH activity (Fig. 3c).

### ATP Maintenance by Exogenous Fatty Acids in Glucose-Deprived Astrocytes

**Fig. 4:**
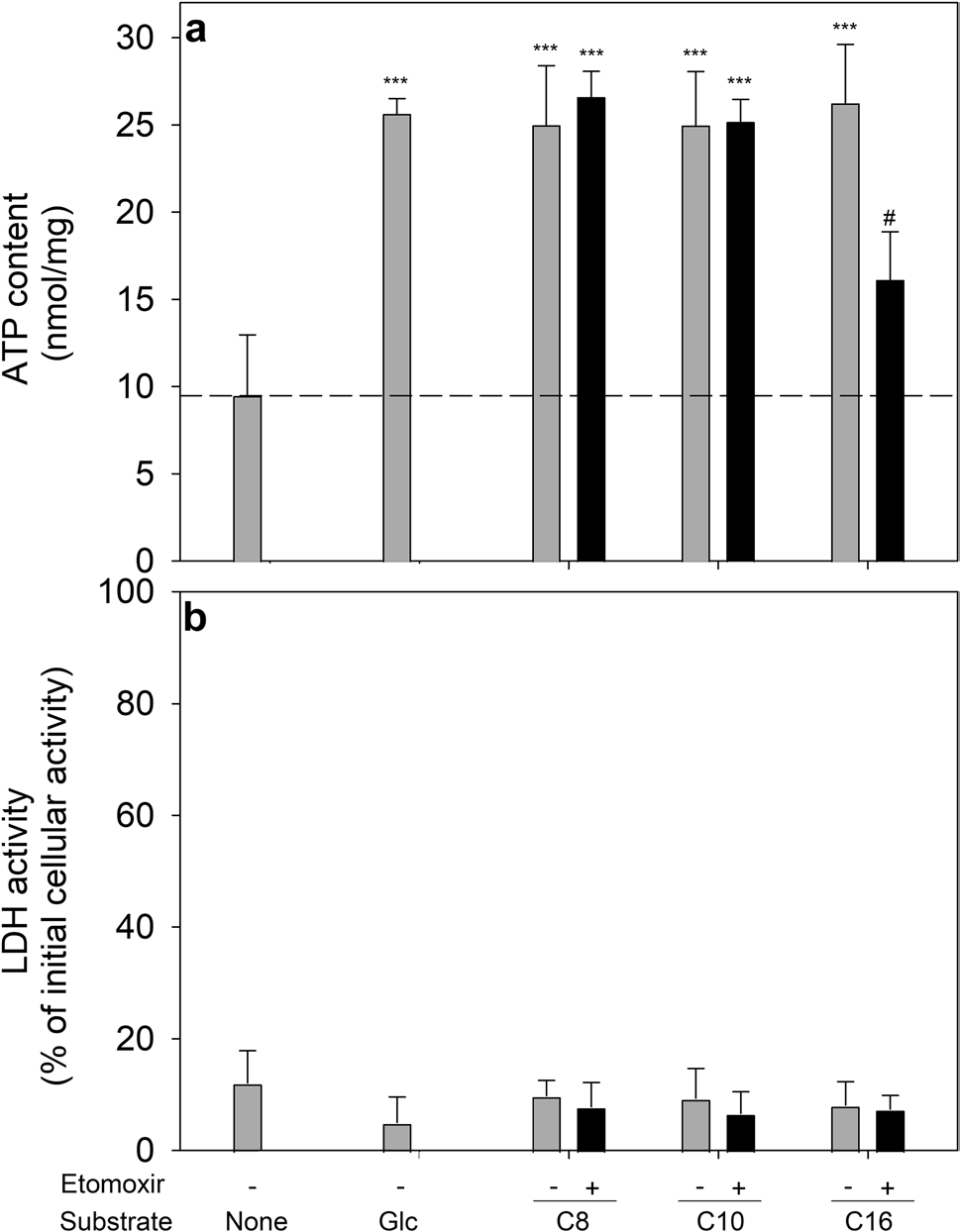
Use of fatty acids as exogenous substrates to maintain a high ATP level in glucose-deprived cultured astrocytes. The cultures were incubated for 24 h in glucose-free incubation buffer or in buffer that had been supplemented with either 5 mM glucose or with 1 mM of the octanoate (C8), decanoate (C10) or palmitate (C16) in the absence or the presence of etomoxir (30 µM) before the specific cellular ATP content (**a**) and the extracellular LDH activity (**b**) were determined. The initial cellular ATP content at the onset of the incubation was 34.2 ± 2.9 nmol/mg, the initial cellular LDH activity was 144 ± 47 nmol/(min × well) and the initial protein content was 144 ± 27 µg/well. The data shown represent means ± SD of values that were obtained in experiments performed on 3 independently prepared cultures. The significance of differences (ANOVA with Bonferroni post hoc test) compared with the data obtained for cells that had been incubated without glucose and fatty acids (None) are indicated by ***p < 0.001. The significance of differences (t-test) of data derived from incubations with or without etomoxir are indicated by ^#^p < 0.05

Extracellular fatty acids are known to be metabolised by astrocytes [15–19]. To test whether the application of fatty acids can prevent the ATP loss in glucose-deprived astrocytes, the medium chain fatty acids octanoate (C8) and decanoate (C10) or the long chain fatty acid palmitate (C16) were applied to the cells in glucose-free buffer for 24 h. In a concentration of 1 mM all three fatty acids prevented the loss in cellular ATP during starvation as good as 5 mM glucose (Fig. 4a), while in the absence of fatty acids (control with fatty acid-free BSA and 1% ethanol) the cellular ATP content had been lowered within 24 h to around 30% of the initial cellular ATP content (Fig. 4a). The ATP maintaining effect of palmitate, but not that of octanoate, decanoate (Fig. 4) or glucose (data not shown), was compromised by etomoxir, an inhibitor of carnitine palmitoyltransferase I (CPT1) [37, 38] (Fig. 4a). None of the conditions investigated caused any obvious impairment of cell viability as indicated by the absence of any increase in extracellular LDH activity (Fig. 4b).

Investigation of the concentration-dependent potential of fatty acids to maintain cellular ATP levels in glucose-deprived astrocytes revealed that palmitate is more potent than octanoate and decanoate to maintain cellular ATP levels (Fig. 1e). The concentration of octanoate that was calculated to provide half-maximal ATP maintenance was almost twice of the concentration calculated for palmitate (Table 2). However, if the concentrations of applied oxidisable carbon atoms were taken into account almost identical concentration-dependencies (Fig. 1f) and half-maximal effect concentrations (Table 2) were found for the three fatty acids investigated (Table 2), which were significantly lower than those calculated for glucose (Table 2).

### Nucleosides as Exogenous Substrates to Maintain ATP Levels in Glucose-Deprived Astrocytes

Purine nucleosides have been reported to prevent ATP depletion and acute toxicity in peroxynitrite-stressed glucose-deprived astrocytes [14]. During a 24 h glucose-deprivation under the experimental conditions used for the current study, the presence of 2 mM of the purine nucleosides adenosine, inosine and guanosine as well as the adenosine phosphates AMP and ADP almost completely prevented the ATP depletion (Table 3). For all nucleosides investigated, the presence of dipyridamole, an inhibitor of nucleoside transport [39, 40], lowered by their potential to maintain cellular ATP levels in glucose-deprived cells by 40–60% (Table 3). None of the conditions investigated caused any obvious impairment of cell viability as indicated by the absence of any increase in extracellular LDH activity (Table 3).

**Table 3:** Maintenance by nucleosides of a high ATP level in glucose-deprived cultured astrocytes

Substrate	Control		Dipyridamole		
	LDH (%)	ATP content (nmol/mg)	LDH (%)	ATP content (nmol/mg)	ATP content (% of control)
None	11 ± 4	8 ± 2***	10 ± 5	8 ± 2***	100 ± 0
Glucose	12 ± 8	28 ± 3	5 ± 6	29 ± 5	104 ± 18
AMP	9 ± 13	30 ± 6	7 ± 5	17 ± 5**^, #^	57 ± 17**
ADP	15 ± 5	23 ± 3	17 ± 11	8 ± 1***^, ##^	35 ± 4***
Adenosine	7 ± 5	38 ± 5	2 ± 3	21 ± 3^##^	55 ± 8***
Inosine	8 ± 5	32 ± 4	5 ± 5	14 ± 1**^, ##^	44 ± 3***
Guanosine	8 ± 8	27 ± 5	17 ± 13	10 ± 1***^, #^	37 ± 4***

The cultures were incubated for 24 h in glucose-free incubation buffer that had been supplemented with 2 mM of the indicated substrates in the absence or the presence of 10 µM dipyridamole before the cellular ATP content and the extracellular LDH activity (in percent of the initial cellular LDH activity) were determined. The initial cellular ATP content at the onset of the incubation was 34.7 ± 3.9 nmol/mg, the initial cellular LDH activity 173 ± 58 nmol/(min × well) and the initial protein content 116 ± 42 µg/well. The data represent means ± SD of values that were obtained in experiments performed on 3 independently prepared cultures. The significance of differences (ANOVA with Bonferroni post hoc test) compared with the data obtained for glucose-treated cells is indicated by**p < 0.01 and ***p < 0.001. The significance of differences (t-test) between values observed for cells that had been treated with and without dipyridamole is indicated by ^#^p < 0.05 and ^##^p < 0.01

## Discussion

Glucose deprivation of cultured astrocytes in an amino acid-free buffer lowers the specific cellular ATP contents within 24 h to around 30% of the initial ATP contents without compromising cell viability, consistent with published data [13]. This experimental paradigm was used to investigate various exogenous substrates for their potential to fuel astrocytic ATP regeneration during glucose deprivation. The specific ATP contents that were maintained during a 24 h incubation with glucose (or other substrates) showed some variations between individual experiments and represented between 70 and 85% of the initial ATP contents. This lowered ATP contents compared to the initial values is most likely caused by the absence of serum during the 24 h incubation as serum has recently been reported to contain components which are required to maintain a maximal ATP content in cultured astrocytes [30].

Extracellular substrates which prevented ATP depletion in glucose-deprived astrocytes are considered to be taken up efficiently and to be metabolised with sufficient velocities to allow mitochondrial ATP regeneration during the long starvation period of 24 h. The inability of an extracellular substrate to maintain a high ATP level suggests that the velocities of uptake and/or cellular metabolism of such an extracellular substrate is insufficient to allow mitochondrial ATP regeneration in glucose-deprived astrocytes under the conditions investigated. However, this does not exclude that such substrates are taken up and metabolised by astrocytes for other important cellular processes rather than for mitochondrial ATP regeneration.

The decline in ATP levels during incubation in glucose- and amino acid-free buffer was prevented by application of glucose in a concentration-dependent manner with half-maximal effects observed for an initial glucose concentration of around 0.5 mM. Under such conditions, glucose is rapidly metabolised by cultured astrocytes and large amounts of lactate are generated and released from the cells [11, 13]. For example, 1 mM glucose was completely metabolised by astrocytes within 6 h, while lactate accumulated extracellularly to a concentration of around 1.7 mM and was subsequently metabolised by the cells almost completely within 24 h [13]. Thus, it is not surprising that exogenous lactate and glucose have an identical potential to maintain ATP levels, if the difference in their carbon numbers is taken into account.

In addition to glucose and lactate, also the presence of pyruvate, acetate and β-hydroxybutyrate was found to prevent the ATP loss in glucose-deprived astrocytes. These monocarboxylates have previously been reported to serve as mitochondrial energy substrates for astrocytes [5, 13, 25]. Studying the concentration-dependencies for the different monocarboxylates revealed that among the investigated mitochondrial substrates acetate, lactate and pyruvate were used by the cells with similar half-maximal effects, if the number of oxidizable carbons was taken into consideration. β-hydroxybutyrate appears to have among the investigated monocarboxylates the lowest potential to maintain a high ATP content in glucose-deprived astrocytes which is likely to be caused by the high K_M_ value of monocarboxylate transporter 1 for β-hydroxybutyrate [41], consistent with the slow consumption of β-hydroxybutyrate by cultured astrocytes [25].

Testing of the 20 proteinogenic amino acids in a high concentration of 3 mM for their potential to prevent ATP depletion in glucose-deprived astrocytes revealed that only the presence of either alanine, aspartate, glutamate, glutamine, lysine or proline was able to maintain a high ATP level for 24 h in glucose-deprived astrocytes. The ability of lysine and proline to maintain ATP levels in astrocytes was unexpected, while efficient uptake and metabolism by astrocytes has previously been reported for alanine [24, 25], aspartate [27, 28], glutamate [6, 26] and glutamine [6, 42]. The concentration-dependencies determined for the six amino acids revealed that lysine had among the amino acids investigated the lowest potential to maintain ATP levels in glucose-deprived astrocytes. Lysine is an essential amino acid that can be taken up by astrocytes [27], but details on the catabolism of lysine in astrocytes has to our knowledge not been reported so far. The complex metabolism of the ketogenic amino acid lysine to mitochondrial acetyl-CoA requires, at least in liver, nine enzymes [43–45] which may explain the rather low potential of lysine to serve as exogenous substrate for ATP regeneration in astrocytes.

Proline was among the extracellular amino acids tested the most potent one. A detailed study of proline metabolism of astrocytes has to our knowledge not been reported so far. For glucose-deprived astrocytes the extracellular concentration of applied proline declined within 24 h from 1 mM to around 0.2 mM, clearly demonstrating that the cells consume extracellular proline. This is consistent with literature data for cultured astrocytes reporting that proline can serve as exogenous substrate for the formation of endogenous glutamate that is subsequently used for synthesis of the antioxidant glutathione [46]. In addition, proline has been reported to stimulate the release of glutamate and glutamine from cultured astrocytes [47] and to modulate central metabolism by acting on hypothalamic astrocytes [48]. Proline catabolism is exclusively localized in mitochondria [49]. Cellular proline catabolism starts with its mitochondrial oxidation to pyrroline-5-carboxylate by the flavin-containing proline dehydrogenase that transfers electrons directly onto ubiquinone in the respiratory chain [50]. The contribution of proline dehydrogenase in the observed ability of proline to prevent the loss in cellular ATP content of glucose-deprived astrocytes is clearly demonstrated by the concentration-dependent ability of the competitive proline dehydrogenase inhibitor THFA [36] to prevent both cellular consumption of extracellular proline and the maintenance of cellular ATP levels in glucose-deprived astrocytes. For further metabolism, the ring in the proline dehydrogenase product pyrroline-5-carboxylate will open up to form glutamate γ-semialdehyde which is oxidized to glutamate [50]. In astrocytes, glutamate will be efficiently transaminated or oxidized to α-ketoglutarate [6, 26] that can supply carbon for oxidative ATP regeneration as intermediate of the citric acid cycle. At least in liver mitochondria, proline was efficiently used as substrate for mitochondrial respiration, especially under conditions that require bypassing complex I of the respiratory chain [50].

Among the different amino acids, only cysteine caused severe toxicity in glucose-deprived astrocytes. The neurotoxic potential of cysteine is well known and has been connected to signaling via the N-methyl-D-aspartate glutamate receptor [51, 52]. For cysteine-treated glioblastoma cells rapid mitochondrial hydrogen peroxide production and reductive stress has recently been reported that was worsened by glucose starvation [53]. Whether such processes may contribute to the observed high toxicity of cysteine in glucose-deprived primary astrocytes remains to be elucidated.

Exogenous medium chain and long chain fatty acids have been reported to be metabolised by astrocytes in culture and in brain slices [15, 16, 18–21]. Therefore, it was hypothesized that exogenous fatty acids may be able to maintain a high ATP content in glucose-deprived astrocytes. The concentration-dependencies for the fatty acids octanoate, decanoate and palmitate revealed that these fatty acids are at least as potent as glucose to prevent ATP depletion under the conditions used. Differences were observed for fatty acids of different carbon content. Octanoate had to be applied in twice the concentration of palmitate to maintain ATP levels, consistent with the doubled number of carbon atoms in palmitate. Etomoxir, an inhibitor of carnitine palmitoyltransferase I [37], partially prevented the potential of palmitate to maintain a high ATP level as this fatty acid is transferred via the carnitine shuttle into mitochondria [54]. In contrast, etomoxir did not lower the ATP maintaining potential of the medium chain fatty acids which is consistent to literature data on the inability of etomoxir to affect the metabolism of these fatty acids in astrocytes [20]. Likely explanation for this finding is that medium chain fatty acids are not transferred by the carnitine shuttle into mitochondria but rather permeate the inner mitochondrial membrane in protonated form and become activated to acyl-CoA in mitochondria [55].

Purine nucleosides such as adenosine, guanosine and inosine as well as AMP and ADP were able to prevent in a high concentration of 2 mM the ATP depletion in glucose-deprived astrocytes. This is consistent with a previous report on the ATP- and viability-maintaining effect of such compounds in peroxynitrite-stressed glucose-deprived astrocytes [14]. However, the concentrations of purine nucleotides needed to prevent ATP depletion in our 24 h starvation paradigm was much higher (around at least 1 mM) than those for the previously reported effects within 6 h in peroxynitrite-treated glucose starved astrocytes (15 µM) [14], suggesting that for efficient maintenance of a high cellular ATP content for an extended incubation period of 24 h high initial amounts of nucleosides are needed. As the utilization of all nucleosides investigated was at least partially inhibited by dipyridamole, an inhibitor of the equilibrating nucleoside transporter 1 [39, 40] it can be assumed that an intracellular utilization of purine nucleosides is involved in the observed ATP maintaining effects, consistent with literature data [14]. Also the ability of extracellular ADP and AMP to prevent cellular ATP loss was prevented by the presence of dipyridamole, suggesting that these adenosine phosphates are hydrolysed extracellularly by astrocytic ectohydrolases [39, 56] to adenosine which is subsequently taken up into the cells and used to prevent cellular ATP loss. An enzyme which may be involved in the cellular utilization of nucleosides for maintaining ATP levels is purine nucleoside phosphorylase. This enzyme is present in high activity in cultured astrocytes [57] and catalyses the phosphorolysis of a nucleoside to the free purine base plus ribose-1-phosphate. The latter could be further metabolised for energy production via glycolysis and/or mitochondrial respiration.

In conclusion, the experimental paradigm of a 24 h glucose starvation was used to investigate the potential of various exogenous substrates to be used by cultured astrocytes as mitochondrial fuels to maintain a high cellular ATP content. Monocarboxylates, six amino acids as well as fatty acids and purine nucleosides were identified as exogenous substrates that can be used by astrocytes to keep their ATP concentration high during glucose deprivation. Thus, astrocytes have sufficient transport and metabolic capacity to make use of the carbon of the respective exogenous substrates to efficiently fuel mitochondrial ATP regeneration. Considering the content of oxidisable carbon in the different extracellular substrates applied revealed that proline and fatty acids are more potent that glucose and monocarboxylates to prevent ATP loss in starved astrocytes.

The 24 h aglycemic condition that was applied in our study does not reflect an in vivo situation, but rather allowed to investigate the potential of astrocytes to take up and metabolise a single applied exogenous substrate. In vivo, a mixture of both endogenous and exogenous substrates will be available in brain cells to prevent an ATP loss in glucose-limited hypoglycemic conditions. Hypoglycemia is a common feature for many human disease conditions ranging from insulin-induced hypoglycemia of neonates, via diabetes to stroke and neurodegeneration [58–60]. To test for the preferred energy substrates in relation to in vivo conditions, mixtures of exogenous substrates in the concentrations present in brain or cerebrospinal fluid, could be tested for their potential to maintain a high ATP content in brain cells under aglycemic or hypoglycemic conditions. In addition, it remains to be elucidated whether the metabolic repertoire that we found for cultured primary rat astrocytes is also available to human astrocytes.

Unexpected was the finding that proline has a high potential to maintain already in micromolar concentrations a high ATP content in glucose-deprived cultured astrocytes. The sources of extracellular proline in brain that could be metabolised by astrocytes remains to be identified. The concentration of proline in human serum has been reported to be in the higher micromolar range (170 µM [61], 450 µM [62]), while only 6 µM proline have been determined for cerebrospinal fluid [61], suggesting that peripheral proline may be one proline source for astrocytes. Further studies are now required to elucidate in detail the proline metabolism of astrocytes and of other brain cells, especially considering that proline has well known behavioral and neurochemical effects [63] and that the proline metabolism is strongly connected to neurological and psychiatric disorders [49].

## Data Availability

Enquiries about data availability should be directed to the authors.
